# Complete Suppression of the Gut Microbiome Prevents Acute Graft-Versus-Host Disease following Allogeneic Bone Marrow Transplantation

**DOI:** 10.1371/journal.pone.0105706

**Published:** 2014-09-02

**Authors:** Jaak M. Vossen, Harry F. L. Guiot, Arjan C. Lankester, Ann C. T. M. Vossen, Robbert G. M. Bredius, Ron Wolterbeek, Hanny D. J. Bakker, Peter J. Heidt

**Affiliations:** 1 Department of Pediatrics, Leiden University Medical Center, Leiden, The Netherlands; 2 Department of Medical Microbiology, Leiden University Medical Center, Leiden, The Netherlands; 3 Department of Medical Statistics and Bioinformatics, Leiden University Medical Center, Leiden, The Netherlands; 4 Biomedical Primate Research Centre, Rijswijk, The Netherlands; Charité-University Medicine Berlin, Germany

## Abstract

The hypothesis that elimination of facultative and strict anaerobic microorganisms from the gastro-intestinal tract by antimicrobial drugs in the period of time around allogeneic bone marrow transplantation (BMT) prevents acute graft-versus-host disease (GVHD), was examined in a cohort of 112 children grafted between 1989 and 2002 for hematological malignancies. All patients received T-cell replete marrow from human leukocyte antigens (HLA) matched sibling donors under identical transplantation conditions. To eliminate microorganisms from the gastro-intestinal tract, total gastro-intestinal decontamination (GID) was applied by high doses of non-absorbable antimicrobial drugs while the graft recipient was maintained in strict protective isolation. About half of the children (51%) proved to be successfully decontaminated, and about half (49%) unsuccessfully. One recipient got acute GVHD in the first group and 8 in the second group (p = 0.013). The degree of success of total GID was decisive for the occurrence of acute GVHD, irrespective of the presence of other risk factors such as higher age of recipient and/or donor, female donor for male recipient and carriership or reactivation of herpesviruses. Our results demonstrate that successful total GID of the graft recipient prevents moderate to severe acute GVHD. We suppose that substantial translocation of gastro-intestinal microorganisms or parts of these, functioning as microbial-associated molecular patterns (MAMP's), triggering macrophages/dendritic cells via pattern recognizing receptors (PRR's) is prohibited. As a consequence the initiation and progression of an inflammatory process leading to acute GVHD is inhibited.

## Introduction

Acute graft-versus-host disease (GVHD) is a major cause of morbidity and mortality in children undergoing allogeneic bone marrow transplantation (BMT). Despite more efficient prevention, e.g. by optimizing human leukocyte antigens (HLA) matching between donor and recipient with high-resolution DNA typing and by improved pharmacological prophylaxis, e.g. with methotrexate (MTX) together with cyclosporin A (CsA), the frequency of ≥ grade II acute GVHD remains between 20 and 30% in children following a T-cell replete allogeneic BMT for a hematological malignancy [1]–[3]. T cell depletion of the graft is an efficient method to avoid the occurrence of acute GVHD; the reverse effect of such graft manipulation is a delayed immune recovery, with a greater risk of opportunistic infections and of relapse of the malignancy. As an alternative for T cell depletion, evidence has been provided in both laboratory animals and humans that complete suppression of the intestinal microorganisms by antimicrobial drugs reduces the occurrence of acute GVHD, besides preventing endogenous bacterial and fungal infections in the immediate post-BMT period [4]–[8].

Acute GVHD is considered a succession of inflammation and donor T-cell activation [9]. The assumed initiators of the inflammatory reaction are whole microorganisms or parts of bacteria and yeasts which may translocate through the mucosal barrier of the intestinal tract as a result of increased permeability after myelo-ablative conditioning of the graft recipient. Based on animal experiments and our earlier retrospective study, we initiated the present study, the aim of which was to evaluate whether successful complete suppression of the intestinal tract microorganisms of the host using total gastro-intestinal decontamination (GID) during the period between the start of conditioning until about one month after allogeneic BMT prevented acute GVHD. For that purpose we analyzed a homogeneous cohort of children with a hematological malignancy who consecutively received a T-cell replete BMT from an HLA-matched sibling donor (MSD) following myelo-ablative conditioning. Also the possible relation between bacterial and fungal infections post-BMT, as well as of the seropositivity for and infection with DNA-viruses and the occurrence of acute GVHD was investigated.

## Patients and Methods

### Patients

All patients were treated according to the protocols and guidelines of the Dutch Childhood Leukemia Study Group (DCLSG) which were approved by the Medical Ethics Committee of the Leiden University Medical Center. Written consent for participation in the DCLSG protocols was obtained from the parents or guardians. Co-consent was obtained from patients ≥12 years of age.

The study cohort consisted of 119 children with a hematological malignancy who were transplanted consecutively with T-cell replete bone marrow from a MSD in the period from January 1^st^, 1989 to January 1^st^, 2002. All patients were similarly treated in strict protective isolation and received non-absorbable antimicrobial drugs orally for total GID (see below), the effect of which was controlled by careful microbiological surveillance. The patients were homogeneous with respect to conditioning, composition of the graft and pharmacological GVHD prophylaxis.

Seven patients were excluded from evaluation, either because of insufficient microbiological data (n = 2) or because they died before 50 days after BMT due to other causes than GVHD (n = 5) (see further). The total number of evaluable cases thus was 112. The characteristics of these patients are given in Table 1.

**Table 1:** Characteristics of the BMT recipients and donors.

Total number of recipients	112
Recipient age at BMT (yrs.)	
median (range)	8.0 (0.5–17.3)
0.5–9	71
≥10	41
Recipient gender	
M	72
F	40
Donor age (yrs.)	
median (range)	7.8 (0.6–29.7)
0.6–19	111
≥20	1
Donor gender	
M	58
F	54
Recipient's original diagnosis	
High-risk acute leukemiaa	
ALL CR 1	17
ALL CR 2	28
ALL ≥ CR 3	2
AML CR1	35
AML CR2	3
Chronic myelocytic leukemia	3
JMML	7
Myelo-dysplastic syndrome	12
NHL CR2	5

Abbreviations: ALL =  acute lymphoblastic leukemia; AML =  acute myeloid leukemia; CR =  complete remission; F =  female; JMML  =  juvenile myelo-monocytic leukemia; M =  male; NHL  =  non- Hodgkin lymphoma.

a According to the treatment protocols of the Dutch Childhood Leukemia Study Group.

### BMT procedure

All children were pretreated with a myelo-ablative conditioning regimen according to the then current guidelines of the European Group for Blood and Marrow Transplantation (EBMT). This consisted of a combination of chemotherapy (cyclophosphamide 120 mg/kg IV, total dose, with the addition of cytosin-arabinoside or VP-16 in some cases) and TBI (unfractionated, 7 Gy×1, 7.5 Gy×1, 8 Gy×1 or 6 Gy×2, according to age) in children above 2 years of age; younger children were given busulfan (20 mg/kg orally, total dose) or busulfex (16 mg/kg IV, total dose) instead of TBI, and cyclophosphamide (200 mg/kg IV, total dose). They received a T-cell replete bone marrow graft from a MSD.

GVHD prophylaxis consisted of MTX-short course (10 mg/kg/day IV at days +1, +3 and +6 (and in some cases +11) plus CsA (2 mg/kg/day IV until engraftment, and 6 mg/kg/day orally thereafter until 180 days after BMT) in all patients.

### Gnotobiotic measures and antimicrobial drugs

All graft recipients were nursed in a strict protective environment from 2 weeks before until about 1 month after BMT, using either a laminar down-flow isolator or an ultra- clean room in combination with aseptic nursing techniques and sterilization of food, beverages and all other items brought into the isolation facility, as described before [10]. During consolidation or maintenance chemotherapy for leukemia before admission, most children were given antimicrobials for selective suppression of potentially pathogenic.

Enterobacteriaceae and yeasts (selective GID) and co-trimoxazole for *Pneumocystis jiroveci* prophylaxis. After entering the strict protective isolation facility an inventory of the potentially pathogenic microorganisms, colonizing the skin, nose, throat and gut was made, after which the graft recipients received high doses of non-absorbable antimicrobial drugs orally for the non-selective i.e. total suppression of the gastro- intestinal microorganisms (total GID), from day -10 until day +30 (in some later cases ≥+20) after BMT. The combinations of antimicrobial drugs, administered per os were, besides amphotericin B (2000 mg dd), gentamycin (800 mg dd) plus cefaloridin (2000 mg dd), from 1988 to August 1993 for patients ≥20 kg body weight [11]. Thereafter, first- generation cephalosporins were no longer available and were replaced by second- generation cephalosporins i.e. cefuroxime or ceftriaxone IV in therapeutical dosage or by vancomycin per os (1000 mg dd). From 1995 onwards piperacillin/tazobactam was given per os (900 mg dd) for patients ≥20 kg body weight. The daily doses were divided over 3 to 4 gifts per day. For patients <20 kg body weight half of the daily doses was administered. After discontinuing total GID, recontamination was done by per oral administration of Biogarde (starter culture for dairy products containing.

*Bifidobacterium bifidum*, *Lactobacillus acidophilus* and *Streptococcus termophilus* strains; kindly supplied by Cargill Texturizing Solutions, Deutschland GmbH, Bönen, Germany) and an exclusively anaerobic human intestinal donor flora [12], during 5 days. Ganciclovir or foscarnet was only administered pre-emptively when CMV pp65- antigenemia was detected. Documented infections were treated with appropriate antimicrobial drugs. No systemic antimicrobial drugs were given prophylactically until discharge, after which co-trimoxazole prophylaxis was restarted.

### Microbiological surveillance and documentation of infections

Swabs from nose, throat and (if indicated) skin, and samples from stool were taken twice weekly during the period from 10 days before until 30 days after BMT. The target microorganisms were all facultative anaerobic and strictly anaerobic microorganisms of the gastro-intestinal tract. Therefore, routine bacteriological and mycological cultures from the swabs were performed, and semi-quantitative cultures from the stool samples were made using a serial 1∶10 dilution in brain-heart infusion (BHI) broth, followed by inoculating the dilutions on selective media to isolate and identify facultative anaerobic gut microorganisms, serving as indicators for the effect of GID, i.e. Enterobacteriaceae, *Enterococcus faecalis* and *Candida* spp. The detection limit of this technique was estimated to be 102 miroorganisms/g feces [13]. Also other facultative anaerobic bacteria e.g. *Pseudomonas* spp., coagulase-positive and -negative staphylococci and yeasts, mostly contaminating the intestinal tract, were traced.

Strictly anaerobic bacteria were not cultured as a routine. In order to get an impression of their presence and heterogeneity in the feces, Gram stains were made of all fecal samples and investigated microscopically.

Bacteremia and severe bacterial and fungal organ infections were defined as the combination of signs and symptoms of an infection plus a positive culture of potentially pathogenic bacteria or yeasts using routine microbiological laboratory techniques; for the detection of fungal lesions in organs high-resolution CT (HR-CT) scan was used.

The serostatus of the *Herpesviridae* at the time of BMT was determined in graft recipients and donors by serology for HSV, VZV, CMV and EBV using standard techniques, i.e. HSV IgG and VZV IgG (until 1998) by immunofluorescence, for CMV, EBV and VZV IgG (from 1998) by ELISA. The serostatus was not routinely investigated pre-BMT for HHV-6 and adenoviruses (HAdV) because of the universal presence of these viruses in the human population. In order to trace infection/reactivation of these DNA-viruses, and to allow for pre-emptive treatment with antiviral drugs, following diagnostic tests were done in the recipient at regular intervals. Pp 65-antigenemia in blood leucocytes was determined weekly in case of CMV-seropositivity of either recipient or donor; ≥1 pp 65-positive leucocytes per 50.000 blood leucocytes was the criterium of CMV-infection [14], as was the isolation of the virus from a throat swab or urine culture. HAdV infection was defined as a positive culture of feces, throat swab or urine taken once in 1-2 weeks during the observation period [15]. EBV infection was diagnosed by the detection of EBV antigens in blood lymphocytes in cases with suspicious signs and symptoms such as fever, lymph node swelling and hepato-splenomegaly [16]. Real-time PCR to detect the above-mentioned DNA-viruses and determine their load in blood samples was not yet routinely available during the study period of 1989 to 2002. HSV- or VZV-disease was confirmed by culture of a sample taken from a typical lesion of a mucous membrane or the skin.

### Variables evaluated

The primary goal of this study was to judge whether successful suppression of the intestinal microorganisms by total GID reduced the occurrence of acute GVHD, diagnosed and graded according to standard criteria [17], in the T-cell replete BMT setting. To evaluate the efficacy of total GID, the minimum number of fecal samples to be investigated during the period between day – 10 and +30 had to be 5, of which at least 2 samples had to be obtained before the day of BMT (day 0), and at least 3 after day 0. In 2 cases less than 5 samples were obtained during that period; they were excluded from evaluation (see before). Total GID was considered successful when in the critical period from 10 days before until 30 days after BMT none of the bacterial or fungal species, as mentioned before, could be cultered in ≥3 stool samples [8]. Additional goals of this study were to trace the possible effect on the occurrence of acute GVHD of microbiologically documented severe infections, i.e. bacteremia, fungemia and severe organ infections, taking place between BMT and 50 days after BMT, and of the serological status for *Herpesviridae* of the host and the donor before BMT. Also the possible coherence of infections/reactivations with CMV, EBV and HAdV and of HSV- and VZV-disease with acute GVHD was investigated.

Moreover, other variables, known from clinical studies to possibly affect the occurrence of acute GVHD, such as age of donor and recipient and female donor for male recipient [18] were included in the evaluation.

### Statistical analysis

All statistical analyses were carried out by the IBM-SPSS 20 statistical package. P-values less than 0.05 were considered as statistically significant. Kaplan-Meier analyses with log-rank tests were used for categorical predictors. The occurrence of acute GVHD and relapse-free survival (RFS) and overall survival (OS) after transplantation were analyzed by Kaplan-Meier curves with log-rank tests. For OS, death was the event of interest, and for RFS, relapse of the malignancy or death. Five-year survival rates for OS and RFS were compared with the Z-test for normal distributions. Possibly confounding variables such as age of donor and recipient and female donor for male recipient within the two groups (successful vs. unsuccessful) of total GID, were compared with median tests and χ^2^ tests. The evaluation cut-off point was January 1^st^, 2003. The median follow- up of survivors was 3.90 years (range 0.16–13.73).

## Results

### Success or failure of total GID

The median number of stool samples, investigated microbiologically during the period of −10 to +30 days, was 9 (range 5–13) per individual patient. Total GID was successful, according to the criteria described in the methods, in 57/112 (51%) graft recipients and was a failure in 55/112 (49%) graft recipients. The microorganisms that caused these failures were *Candida* spp. (n = 32), coagulase negative staphylococci (n = 7), *Enterococcus faecalis* (n = 5), and a combination of 2 of these microorganisms (n = 9); in 2 patients *Pseudomonas* spp. persisted together with one or more of the formerly mentioned microorganisms. Systemic treatment with fluconazole, or an other azole-drug, for the elimination of persisting *Candida* spp. from the gastro-intestinal tract was only effective in a quarter of the colonized graft recipients.

### Relation between effectiveness of total GID and occurrence of acute GVHD

**Figure 1:**
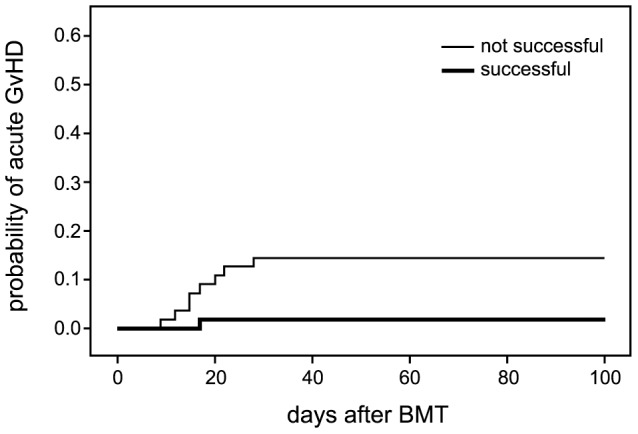
Frequency of acute GVHD (any grade) following BMT in successfully and not successfully decontaminated children with hematologic malignancy (p = 0.013).

The number of graft recipients that developed acute GVHD after BMT was very low, i.e. _9/112_ (8%). Eight children developed grade I GVHD according to the criteria of Przepiorka et al. (17), i.e. only involvement of the skin. One patient got grade II (skin stage 2, gut stage 1, and liver stage 2). None of the patients died as a result of acute GVHD. The cumulative probability of acute GVHD in relation to the success or failure of total GID is shown in Figure 1.

Successful total GID resulted in significantly less acute GVHD (p = 0.013; log-rank test). The only case with grade II GVHD occurred in the group of patients with unsuccessful total GID.

### Severe bacterial and fungal infections and possible relation with occurrence of acute GVHD

A total of 12 severe infections were observed in 9 graft recipients; 6 patients had one infectious episode and 3 patients experienced 2 infectious episodes (see Table 2). When restricting the documentation of infections to the period of total GID and strict protective isolation, i.e. from day 10 before to day 30 after BMT, the number of infections was 9, of which 6 occurred in patients with a failure of total GID and 3 in patients with successful total GID. In one patient from whom a coagulase negative *Staphylococcus* was isolated from the blood, the total GID was a failure due to the persistence of this microorganism in the stool. In the other cases with a severe infection the causative microorganism could be cultured once or more from one of the sampling sites within the period between BMT and 30 days after BMT with the exception of *Bacillus cereus* which was only cultured from the blood once. None of the severe infections was lethal. One out of 4 patients with failed total GID and severe infection got grade I acute GVHD versus none out of 5 with successful total GID and a severe infection. Altogether, no correlation was found between severe bacterial and fungal infections and the occurrence of acute GvHD.

**Table 2:** Severe bacterial and fungal infections after BMT and relation to acute GVHD.

Microorganism^a^	site/organ	n positive cultures	day after BMT^c^	S/F of TGID^d^	acute GVHD^e^
Streptococcus mitis*	blood	1	7	F	0
Escherichia coli*	blood	1	7		
Ps. aeruginosa**	urine	1	28	F	0
Streptococcus mitis**	blood	1	8		
Coagulase neg. Staph.	blood	3	12	F	I/22
Bacillus cereus	blood	1	7	F	0
Coagulase neg. Staph.	blood	3	47	S	0
Ps. aeruginosa***	blood	3	46	S	0
Salmonella group B***	feces	3	39		0
Coagulase neg. Staph.	blood	6	10	S	0
Aspergillus spp.	lungsb	1b	16	S	
Staph. aureus	blood	1	8	S	0

a in case only one blood culture was positive for coagulase negative Staphylococcus (which occurred 10 times) this was considered to be a contamination.

b diagnosed by HR-CT scan.

c (first) day of positive culture.

d S/F of TGID: success or failure of total gastrointestinal decontamination.

e grade and day of start of GVHD.

*, **, ***single patients with 2 infectious episodes.

### HSV, VZV, CMV and EBV serostatus in recipients and donors before BMT and infection/reactivation of DNA-viruses, or disease with DNA-viruses after BMT in relation to occurrence of acute GVHD

**Figure 2:**
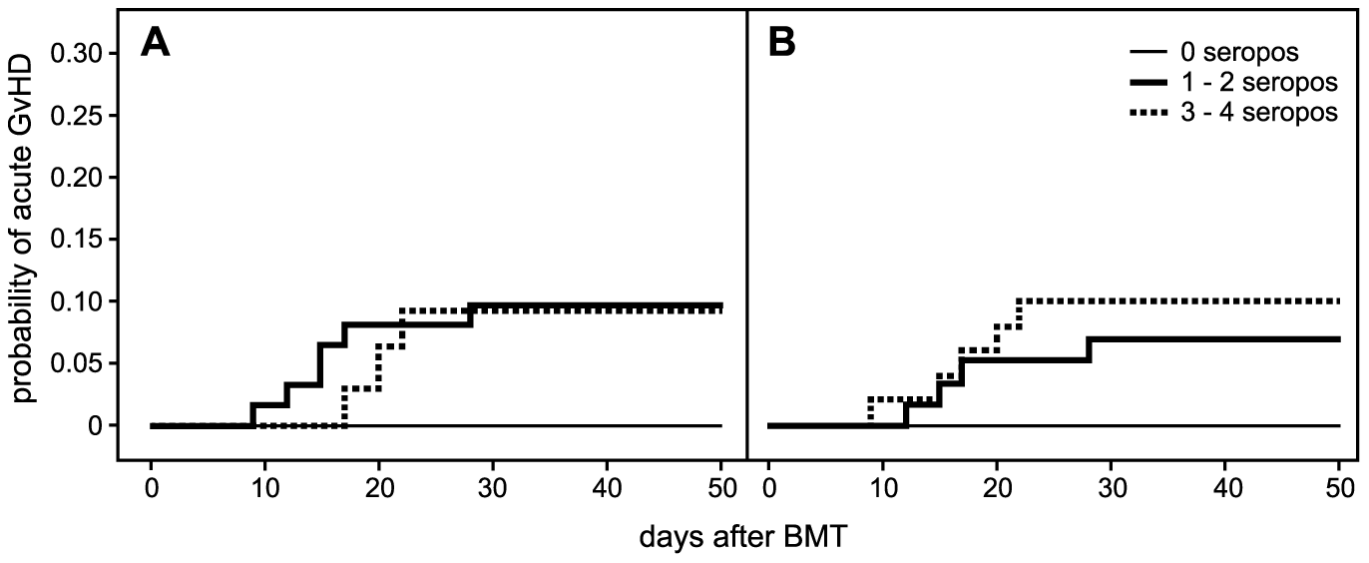
Frequency of acute GVHD (any grade) in relation to serostatus for herpesviruses of donor (A) and recipient (B) following BMT (p = 0.404 and p = 0.706, respectively).

The serological status for HSV, VZV, CMV and EBV of graft recipients and their donors is given in Table 3. There is no correlation between the negative serostatus of the BMT couples versus other serostatus combinations and the occurrence of acute GVHD. Also seropositivity for increasing numbers of herpesvirus types, both in the graft recipient and the donor, did not augment the risk for acute GVHD (Figure 2). As far as could be observed all infections were reactivations of DNA-viruses carried by the host. EBV-related disease did not occur in this patient population. The other DNA- viruses reactivated (CMV, HAdV) or caused disease (HSV, VZV) 44 times during the observation period from BMT to day 50 after BMT (Table S1). Because some patients suffered from more than one virus reactivation or disease, the total number of cases was 39. Of these 39 patients, 5 got acute GVHD versus 4 out of 73 cases without virus reactivation (p = 0.173; χ^2^ test). Thus, reactivation of or disease with endogenous DNA-viruses was not associated with an increased risk for acute GVHD.

**Table 3:** HSV-, VZV-, CMV- and EBV- serological status of donor and recipient before BMT and relation to acute GVhD at day 50 post BMT.

Virus	serostatus	n	acute GVHD	p-value^c^
HSV	D–R–a	50	4 (1)^b^	
	D+R–	9	0	
	D–R+	32	2	0.596
	D+R+	21	3	
VZV	D–R–	13	0	
	D+R–	13	0	
	D–R+	14	1	0.377
	D+R+	72	8 (1)	
CMV	D–R–	73	5 (1)	
	D+R–	7	1	
	D–R+	12	1	0.900
	D+R+	20	2	
EBV	D–R–	23	2 (1)	
	D+R–	9	1	
	D–R+	27	1	0.826
	D+R+	53	5	

a DR: donor c.q. recipient of BMT

b ( ): number of ≥ grade II acute GVHD

c p-value for D-R- versus other combinations; log-rank test.

### Other transplant-related variables possibly influencing the occurrence of acute GVHD

Transplant-related variables found in different studies to affect the occurrence and severity of acute GVHD adversely are increasing age of donor and recipient and female donor for male recipient [18]. Also in this study, using univariate analysis and χ^2^ test, a significant lower frequency of acute GVHD was found in younger patients than in older patients, and a significant higher frequency of acute GVHD occurred in the combination female donor to male recipient versus other combinations (Table S2). These variables may confound the results of the success or failure of total GID on the occurrence of GVHD. Because the number of events (9 acute GVHD in 112 cases) was too small for multivariate analysis, the potential for confounding was assessed by comparing the distribution of these variables over the two total GID groups, i.e. successful versus unsuccessful, using a non- parametrical test (independent samples median test) for the variable age and the Pearson's χ^2^ test for the variable sex of donor and recipient (Table 4). No statistically significant difference of the distribution of possibly confounding variables over the two total GID groups was found.

**Table 4:** Distribution of possibly confounding transplant-related variables within two subgroups of patients.

Parameters^a^	total n	success of TGID	failure of TGID	p-value^c^
Recipient's age ≥8.0^b^	56	25	31	0.282
Donor's age ≥7.8^b^	56	26	30	0.450
F donor M recipient	41	20	21	0.734

a possibly having an adverse effect on the occurrence of acute GVHD.

b ≥ median age.

c see text.

### Transplant-related mortality, relapse-free survival (RFS) and overall survival (OS)

Five patients died shortly after BMT, i.e. between day +18 and +43 post-BMT. The causes of death were lethal Stevens-Johnson syndrome (n = 1), capillary leakage + veno- occlusive disease (VOD) (n = 3) and invasive aspergillosis (n = 1). They were excluded from evaluation of the effect of total GID on acute GVHD. The RFS and OS were evaluated in the 112 graft recipients included in this study: 48 patients relapsed of their disease before January 1^st^, 2003; 47 patients died, 43 as a result of recurrence of their disease and 4 due to transplantation related problems other than GVHD. RFS at 5 years post-BMT was 52.3% for successfully decontaminated patients and 61.3% for not successfully decontaminated patients (p = 0.356; Z-test for normal distributions), and OS at 5 years post-BMT was 52.4%, respectively 63.1% (p = 0.267; Z-test for normal distributions).

## Discussion

Absence or elimination of the intestinal microorganisms prevents infections and acute GVHD, even in histoincompatible transplants. This was documented in germfree and decontaminated mouse models, [4], [5], [19], a beagle model [6] and a rhesus monkey model [7]. The first studies of the possible effect of GID in man were performed in children (retrospective) [20] and adults (prospective) [21], transplanted for severe aplastic anemia. GID had a clear beneficial effect on the frequency of acute GVHD in these patients. From retrospective studies by the Essen BMT-group [22] and our group [8] suppression of the strictly anaerobic gut microorganisms together with the facultative anaerobic gut microorganisms, i.e. total GID, was superior in reducing acute GVHD as compared to selective GID, leaving many strictly anaerobic strains intact. The Seattle BMT-group did not succeed in reproducing such a beneficial effect when grafting adult patients with leukemia [23], but compliance with oral non-absorbable antimicrobial drugs was low and the effect of GID on the quality and quantity of the intestinal microorganisms was not investigated. The Essen BMT-group compared in a prospective open-label study two regimens of antibiotics for decontamination of adult BMT patients with a hematological malignancy. They found significantly less ≥ grade II acute GVHD in the patients whose strictly anaerobic gut flora was also suppressed, besides the facultative anaerobic gut flora [24].

The study described here is a retrospective evaluation of the effect of successful versus unsuccessful GID on the occurrence of acute GVHD in a homogeneous cohort of children, consecutively treated with an allogeneic T-cell replete BMT for a hematological malignancy. The division into two arms to be compared was done *ex post* on grounds of success or failure of total GID. Our data demonstrate that successful suppression of facultative and strictly anaerobic bacteria, and yeasts in the gut prevents the occurrence of acute GVHD. It supports our long-standing hypothesis that the most prominent role in initiating the process leading to acute GVHD is played by intestinal bacteria and yeasts or their components [11], which translocate following damage of the intestinal barrier caused by the conditioning [25]–[28]. These components are recognized as microbial-associated molecular patterns (MAMP's) by pattern recognizing receptors (PRR's) (e.g. TLR's) on macrophages/DC's and initiate an inflammatory process in the gut [29]–[31].

In our setting of BMT under gnotobiotic conditions, i.e. total GID in strict protective isolation, severe infections with bacteria and yeasts are rare [8]. No relationship was found between severe infections and the occurrence of acute GVHD. Literature data on a possible relationship between carriage of herpesviruses by donor or recipient and the occurrence and severity of acute GVHD, are ambiguous [32]–[35].

Although there is some indication of a relationship between seropositvity for an increasing number of herpes virus types and more acute GVHD (see Figures 2A and 2B), and strikingly, the absence of acute GVHD in either seronegative donors or recipients, the differences are not significant. Despite that the question may be raised whether the relationship between increasing age and increasing frequency of GVHD [18], as also found in this study, is not merely a reflection of an amplified stage of T-cell priming by an accumulating carriership of DNA-viruses, associated with increased alloreactivity [36]. Reports on a possible relation between virus infections/reactivations and acute GVHD are controversial [37], [38]. Some persistent DNA- viruses reactivate and may cause disease early, i.e. within 50 days after T-cell replete BMT, e.g. HHV6, HSV and to some extent CMV. Others, e.g. CMV and VZV do so later. HAdV and EBV do not cause disease in the T-cell replete BMT setting, when no serotherapy is used [15], [16]. With the restriction that we did not use RT-PCR technology routinely in our patient population, a causative relationship between virus infections/reactivations and acute GVHD was not found in this study. The opposite seems to be the case: viral infections/reactivations and disease occur at the nadir of immune competence, including during GVHD and its treatment [39].

In conclusion, our study supports the hypothesis that successful suppression of the intestinal microbiome of the bone marrow graft recipient from 10 days before until 30 days after BMT prevents the occurrence of ≥ grade II GVHD. Our results indicate that even unsuccessful total GID may have a beneficial effect on the occurrence of acute GVHD. This should be further investigated with the recently developed molecular biological technology to study the composition of the intestinal microbiome.

**Figure 3:**
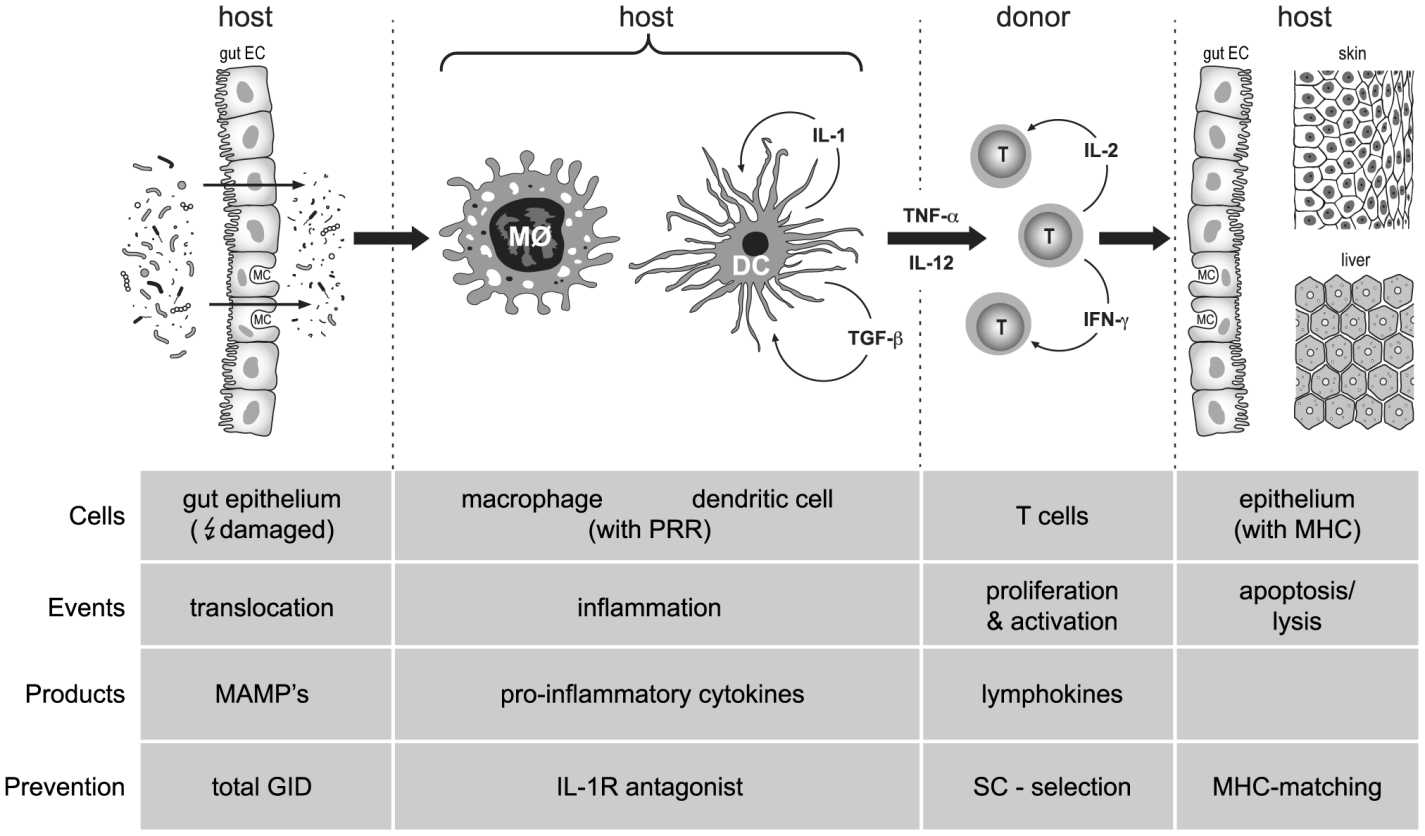
Schematic representation of sequential events, cells and products concerned with acute GVHD following allogeneic BMT. DC, EC, MC,MØ, SC: dendritic cell, epithelial cell, microfold cell, macrophage, stem cell. IFN, IL, TGF, TNF: interferon, interleukin, T-cell growth factor, tumor necrosis factor. MAMPS, MHC, PRR: microbial-associated molecular patterns, major histocompatibility complex, pattern recognizing receptor.

Figure 3 depicts the chain of events that will result in the destruction of epithelial cells, defined as acute GVHD. It is adapted, as a result of our findings, to the circle of phases according to Ferrara cs. [9]. The fixed sequential events are: 1) translocation of microbial components in the gastro-intestinal tract following damage (conditioning), 2) inflammation with secretion of pro-inflammatory cytokines by macrophages/DC's, 3) activation of lymphocytes (proliferation, production of cytokines) and 4) recognition and destruction of target cells by donor-derived T lymphocytes. In our opinion peptidoglycans, present in the cell wall of strictly anaerobic and facultative anaerobic bacteria and yeasts, are the obvious instigators of the process [40]. Following translocation through the gut epithelium, the barrier function of which has been damaged by the conditioning, their MAMP's are recognized by receptors on macrophages/DC's and they stimulate these cells to produce pro-inflammatory cytokines. There is no evidence from experimental data that LPS plays a role in triggering GVHD; LPS rather amplifies GVHD by increasing cytokine release [41]. Daily oral feeding of different (increasing) dosages of endotoxin to germfree mouse recipients of H2-incompatible bone marrow cells from the day of transplantation until day 30 after transplantation did not result in GVHD (P.J. Heidt, unpublished observation), while also systemic administration of endotoxin to recipients in a murine model of BMT did not aggravate the process of GVHD [41]. Polymorphisms of PRR's and intracellular molecules recognizing microbial determinants such as Toll-like receptors (TLR's) and NOD-like receptors (NLR's) may modulate the severity of the inflammation, as described in GVHD [42]–[44] and in other inflammatory intestinal diseases.

Figure 3 also shows the possible preventive or therapeutical measures that may be taken to reduce the risk of occurrence or worsening of acute GVHD. When leaving T cells in the graft, which has an advantage for immune recovery, and possibly suppresses recurring leukemia, total GID seems the most powerful measure because it may prevent, in a harmless way, the ignition of the inflammatory process leading to GVHD. Spacing TBI and BMT may reduce GVHD mortality as shown in experimental H2-incompatible mouse models [45]. Also tempering of the inflammation, e.g. by blockade of IL-1 or TNF-α may theoretically be effective in controlling acute GVHD [46], but pre-emptive use of IL-1 antagonist in a prospective randomized clinical trial did not show an advantage over placebo [47] and probably needs further adjustments.

Suppression of the intestinal microbiome seems to be an independent factor in GVHD prophylaxis, irrespective of any other possible risk factors, including selection of the donor on the basis of age, sex and serostatus of persistent viruses. Further studies of the intestinal microbiome, e.g. with ribosomal DNA sequencing [48], may throw more light on relevant microbial substances for the ignition of the GVHD process.

## Supporting Information

Table S1 **Infection with HSV, VZV, CMV and HAdV after BMT and relation to acute GVHD.**
(DOCX)

Table S2 **Relationship between possibly confounding transplant-related variables and acute GVHD.**
(DOCX)
