# Supplementary material for: Complete Suppression of the Gut Microbiome Prevents Acute Graft-Versus-Host Disease following Allogeneic Bone Marrow Transplantation
Source: PLoS One. 2014 Sep 2;9(9):e105706. doi: 10.1371/journal.pone.0105706 (PMC4152127; doi:10.1371/journal.pone.0105706)
Supplement: Table S1 — Infection with HSV, VZV, CMV and HAdV after BMT and relation to acute GVHD. (DOCX) [file pone.0105706.s001.docx]

**Table S1: Infection with HSV, VZV, CMV and HAdV after BMT and relation to acute GVHD**

| Virus | | Infection/acute GVHD | | | day after BMT^a^ |
| --- | --- | --- | --- | --- | --- |
|  | no/no | no/yes | yes/no | yes/yes | virus inf. / aGVHD^a^ |
| HSV | 99 | 9 | 9 | 3 | 22/22; 15/17; 11/25 |
| CMV | 84 | 8 | 22 | 2 | 34/22; 22/20 |
| VZV | 107 | 11 | 2 | 1 | 27/15 |
| HAdV | 102 | 10 | 4 | 1 | 27/22 |

^a^ (first) day of virus/antigen isolation in the cases with acute GVHD, and day of start acute GVHD
