# Supplementary material for: Complete Suppression of the Gut Microbiome Prevents Acute Graft-Versus-Host Disease following Allogeneic Bone Marrow Transplantation
Source: PLoS One. 2014 Sep 2;9(9):e105706. doi: 10.1371/journal.pone.0105706 (PMC4152127; doi:10.1371/journal.pone.0105706)
Supplement: Table S2 — Relationship between possibly confounding transplant-related variables and acute GVHD. (DOCX) [file pone.0105706.s002.docx]

**Table S2: Relationship between possibly confounding transplant-related variables and acute GVHD**

| acute GVHD | | | | |
| --- | --- | --- | --- | --- |
| Variables | n | yes | no | p-value^b^ |
| Recipient’s age ⩾ 8.0^a^ | 56 | 8 | 48 | 0.015 |
| Recipient’s age < 8.0 | 56 | 1 | 55 |  |
| Donor’s age ⩾ 7.8^a^ | 56 | 6 | 50 | 0.297 |
| Donor’s age < 7.8 | 56 | 3 | 53 |  |
| F donor, M recipient | 41 | 6 | 35 | 0.051 |
| other D-R^c^ combinations | 71 | 3 | 68 |  |

^a^ ⩾ or < median age

^b^ χ^2^ test

^c^ D-R: donor – recipient of BMT
